# Thromboprophylaxis with enoxaparin is associated with a lower death rate in patients hospitalized with SARS-CoV-2 infection. A cohort study

**DOI:** 10.1016/j.eclinm.2020.100562

**Published:** 2020-10-05

**Authors:** Filippo Albani, Lilia Sepe, Federica Fusina, Chiara Prezioso, Manuela Baronio, Federica Caminiti, Antonella Di Maio, Barbara Faggian, Maria Elena Franceschetti, Marco Massari, Marcello Salvaggio, Giuseppe Natalini

**Affiliations:** a Department of Anesthesia and Intensive Care, Fondazione Poliambulanza Hospital, via Bissolati, 57, Brescia 25124, Italy; b Department of Intensive Care Medicine and Anaesthesiology, Fondazione Policlinico Universitario A. Gemelli, Università Cattolica del Sacro Cuore, Rome, Italy

**Keywords:** Coronavirus, SARS-CoV-2, COVID-19, Heparin, Low molecular weight heparin, Thromboprophylaxis

## Abstract

**Background:**

Severe Acute Respiratory Syndrome CoronaVirus 2 (SARS-CoV-2) infection is associated with hypercoagulability caused by direct invasion of endothelial cells and\or proinflammatory cytokine release. Thromboprophylaxis with enoxaparin is recommended by current guidelines, but evidence is still weak. The aim of this study was to assess the impact of thromboprophylaxis with enoxaparin on hospital mortality in patients admitted for Coronavirus disease 2019 (COVID-19). The effects of enoxaparin on intensive care admission and hospital length-of-stay were evaluated as secondary outcomes.

**Methods:**

Observational cohort study, with data collected from patients admitted to Poliambulanza Foundation with positive real time reverse transcription polymerase chain reaction (RT-PCR) for SARS-CoV-2 from 20th February to 10th May 2020. Multivariate logistic regression with overlap weight propensity score was used to model hospital mortality and intensive care admission, hospital length-of-stay was analyzed with a multivariate Poisson regression. Seven hundred and ninety nine (57%) patients who received enoxaparin at least once during the hospitalization were included in the enoxaparin cohort, 604 (43%) patients who did not were included in the control cohort.

**Findings:**

At the adjusted analysis enoxaparin was associated with lower in-hospital mortality (Odds Ratio 0·53, 95% C.I. 0·40–0·70) compared with no enoxaparin treatment. Hospital length-of-stay was longer for patients treated with enoxaparin (Incidence Rate Ratios 1·45, 95% C.I. 1·36–1·54). Enoxaparin treatment was associated with reduced risk of intensive care admission at the adjusted analysis (Odds Ratio 0·48, 95% C.I. 0·32–0·69).

**Interpretation:**

This study shows that treatment with enoxaparin during hospital stay is associated with a lower death rate and, while results from randomized clinical trials are still pending, this study supports the use of thromboprophylaxis with enoxaparin in all patients admitted for COVID-19. Moreover, when enoxaparin is used on the wards, it reduces the risk of Intensive Care Unit admission.

**Research in context**

**Evidence before this study**

Manifestations of hypercoagulability are common in patients affected by the new Coronavirus disease 19 (COVID-19), and are associated with acute inflammatory changes and laboratory alterations. Hypercoagulability is probably caused by endothelial injury, due to a direct invasion of endothelial cells by the SARS-CoV-2 virus and\or proinflammatory cytokine release. Thromboembolic events are associated with a poor prognosis. In April 2020, the International Society on Thrombosis and Haemostasis developed guidelines for thromboprophylaxis with enoxaparin for patients with COVID-19. Evidence supporting this recommendation is weak and evidence of a mortality benefit in patients treated with enoxaparin is lacking, mostly due to the fact that COVID-19 is a new disease. Therefore, any data that can support or contradict this recommendation can be useful, especially since randomized clinical trials are lacking.

**Added value of this study**

Treatment with enoxaparin during hospital stay was associated with a lower death rate, therefore it supports the use of enoxaparin in all patients admitted to hospital for COVID-19. Moreover, when enoxaparin is used on the wards, it reduces the risk of Intensive Care Unit admission.

**Implications of all the available evidence**

In light of this study, and the reported increased risk of thrombotic events in COVID-19, all patients admitted to the hospital should receive prophylaxis for venous thromboembolism. Randomized clinical trials are needed, so as to provide more evidence on enoxaparin use in COVID-19. Future research should focus on different types of heparin and on directly acting oral anticoagulants, for example on direct oral anticoagulants, and on their application in COVID-19.

Alt-text: Unlabelled box

## 1 Introduction

Coronavirus disease 2019 (COVID-19) is a new disease, caused by Severe Acute Respiratory Syndrome CoronaVirus 2 (SARS-CoV-2) infection.

Manifestations of hypercoagulability are common, associated with acute inflammatory changes and laboratory alterations [1,2]. Hypercoagulability is probably caused by endothelial injury, due to a direct invasion of endothelial cells by the SARS-CoV-2 virus and\or proinflammatory cytokine release [1,3,4].

The inflammatory reactions cause damage to the microvascular system and an abnormal activation of the coagulation system, that result in a generalised small vessel vasculitis and extensive microthrombosis [5]. Manifestations included venous thromboembolism (VTE), pulmonary embolism, stroke, myocardial infarction, microvascular thrombosis, acute arterial thrombosis [1,4,6]. Thromboembolic events are associated with a poor prognosis [7]. Alteration of the ventilation/perfusion ratio can in part explain the profound hypoxia that characterizes this disease [8].

In April 2020, the International Society on Thrombosis and Haemostasis developed guidelines for thromboprophylaxis with enoxaparin for patients with COVID-19 [9].

Evidence supporting this recommendation is weak, mostly due to the fact that COVID-19 is a new disease. Therefore, any data that can support or contradict this recommendation can be useful, especially since randomized clinical trials are lacking.

The aim of this study was to assess the impact of thromboprophylaxis with enoxaparin on hospital mortality in patients admitted for COVID-19. The effects of enoxaparin on intensive care admission and hospital length-of-stay were evaluated as secondary outcomes.

## 2 Methods

### 2.1 Study design and inclusion criteria

In this observational cohort study, we collected data from patients who had been admitted to Poliambulanza Foundation Hospital, a 600-bed tertiary care hospital located in Brescia (Northern Italy), during the pandemic crisis of SARS-CoV-2 between February 20th and May 10th 2020.

The study protocol was approved by the Ethics Committee of Brescia.

Patients were included in the study if they were admitted to the hospital and if real time reverse transcription polymerase chain reaction from a nasopharyngeal swab or bronchoalveolar lavage resulted positive for SARS-CoV-2. Exclusion criteria were: age less than 18 years, or being still admitted to hospital so that a definitive outcome was not available at the time of analysis.

### 2.2 Data collection

Patients characteristics were recorded, including demographics [age, sex, body mass index (BMI)], and co-morbidities (arterial hypertension, diabetes mellitus). The following data were collected within 24 h of hospital admission: routine laboratory investigations, including complete blood count, C-reactive protein (CRP) and lactate dehydrogenase (LDH); partial pressures of arterial oxygen (PaO_2_) and carbon dioxide (PaCO_2_), PaO_2_/FiO_2_ and serum lactate concentration analyzed from arterial blood samples. Treatment with corticosteroids, azithromycin and\or hydroxychloroquine was recorded. The number of patients admitted to Intensive Care Unit (ICU) and length of hospital stay was recorded. Patients were followed until death or discharge from hospital.

At least 10 events for every variable included in a multiple logistic model are needed to ensure an accurate estimate of the regression coefficient. Therefore, with a predicted hospital mortality of about 25% (350 patients) it was estimated that it would be possible to include up to 35 variables in the multiple logistic regression analysis [10,11].

### 2.3 Exposure

The patients were classified into two cohorts, based on exposure to enoxaparin during hospitalization. The enoxaparin cohort included patients that were treated with enoxaparin during hospital stay; admitted patients that did not receive enoxaparin were included in the no enoxaparin cohort. The prescription of thromboprophylaxis and other treatments was the responsibility of the attending physician.

### 2.4 Outcomes

Hospital mortality was the primary outcome. Secondary outcomes were: admission to intensive care and hospital length of stay.

### 2.5 Statistical analysis

Quantitative variables are presented as median (1st - 3rd quartiles) and factor variables as count (percentage).

Bivariate analysis of the outcome was performed on selected *a priori* variables with Fisher's exact test for factorial variables and Kruskal-Wallis test for continuous ones. Association with treatment received was assessed.

A propensity score for treatment allocation was estimated from a multivariable logistic regression model containing patient age, sex, PaO_2_/FiO_2_, lactate, C-reactive protein, Platelets, ICU admission and treatment with corticosteroids, azithromycin or hydroxychloroquine [12]. The overlap weight propensity score method was then applied to a multivariate logit regression modeling the primary outcome (in-hospital mortality). Odds Ratio with 95% Confidence Interval (OR, 95% C.I.) are reported. The same procedure was used for OR of ICU admission, taking in account only enoxaparin treatment received on the wards. Propensity score was used with a Poisson multivariate analysis to model hospital length of stay and Incident Rate Ratios (IRR) with 95% C.I. was reported. Collinearity was assessed with variance inflation factor (VIF), and variables were excluded from the multivariate model if VIF was greater than 3 and if there was correlation with other explanatory variables (for example PaO_2_\FiO_2_ and PaO_2_). Missing values, in covariates, were assessed and replaced by mean substitution. Comparisons were estimated for the two study cohorts. If a statistical significant association was found, the E-values for the point estimate and the confidence interval limit closer to the null were computed, to assess the possible effect of unmeasured confounders [13]. Sensitivity analyses were planned, one excluding patients admitted to intensive care, one analyzing completed cases and one with nearest propensity score matching for the main outcome of hospital mortality. Two post-hoc analysis were conducted: 1) patients in the enoxaparin cohort were divided according to the daily dose of received enoxaparin (prophylactic ≤ 40 mg a day or therapeutic > 40 mg a day), association with hospital mortality was evaluated by estimating OR with the same method as previously reported. 2) patients in the enoxaparin cohort were divided according to the duration of enoxaparin therapy (for 1–2 days, for 2–4 days and for more than 4 days), OR was estimated with the same method as previously reported. Significance was evaluated at α = 0·05 and all testing was 2-sided.

Statistical analysis was performed using R Studio software version 4.0.0 (R Core Team, 2014) and packages ‘psw’ and ‘E-value’ for Overlap weight propensity score and calculation of E-value, respectively.

## 3 Results

**Fig. 1:**
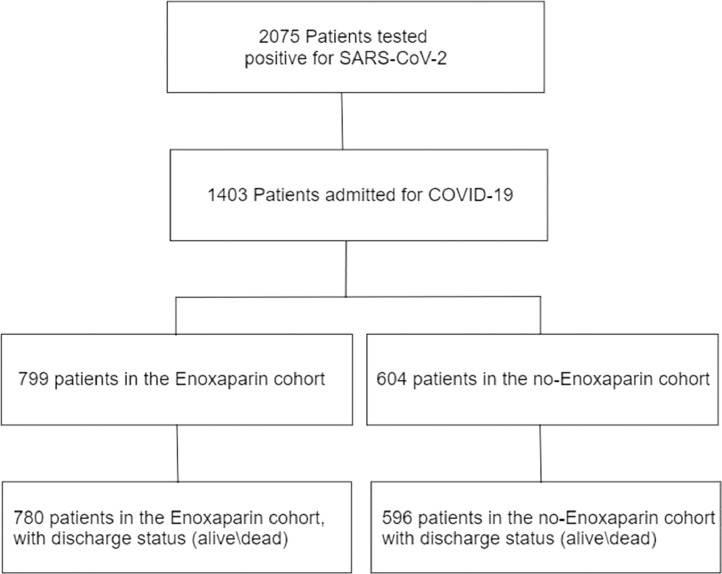
Flowchart of patients included in the study. Severe Acute Respiratory Syndrome CoronaVirus 2 (SARS-CoV-2), Coronavirus disease 2019 (COVID-19).

From 20th February to 10th May 2020, 11,671 patients were admitted to the Emergency Department of our hospital and, among them, 2075 tested positive for SARS-CoV-2, of those 1403 were admitted to hospital. Seven hundred ninety nine (57%) patients received enoxaparin at least once during the hospitalization (enoxaparin cohort) and 604 (43%) patients did not (no enoxaparin cohort). Twenty-seven patients were excluded from the analysis, one because he was younger than 18 years, while the other 26 were still admitted to the hospital and, because of this, the discharge status (alive\dead) was not available at time of analysis (Fig. 1).

Characteristics of patients in the enoxaparin cohort and in the control cohort are presented in Table 1. The median dose of enoxaparin was 40 (40–80 mg) per day and the duration of therapy was 6 (3–9) days. The first dose was administered 1 day (0–3) after hospital admission. Patients in the enoxaparin cohort were older (*p* = 0·01) and mostly male (*p* = 0·04). Patients with a higher BMI were more likely to receive enoxaparin (*p* = 0·001). No statistically significant differences in comorbidities were noticeable between the two cohorts. Patients who did not receive enoxaparin had a significantly lower platelet count (*p*<0·001). D-dimer level on the first day of hospital admission was measured in 290 patients: it was 1534 (603–2437) ng/ml in the enoxaparin group and 1559 (801–3863) ng/ml in the no-enoxaparin group (*p* = 0.28). Survivors had lower concentration of D-dimer than non-survivors, 1362 (634–2627) and 4676 (1499–20,000) ng/ml, respectively (*p*<0·001). Fifty three (8·8%) patients in the no enoxaparin group and 13 (1·6%) in the enoxaparin group were already on chronic oral anticoagulation before being admitted to the hospital.

**Table 1:** Patients characteristics of enoxaparin and control cohorts.

		No Enoxaparin treatment	Enoxaparin	*p* value
Patients	N (%)	604 (43)	799 (57)	
Age	years	72 (59·8–80)	69 (60–77)	0·01
BMI	kg*m^−2^	26 (23–28)	26 (24- 29)	0·001
Male	N (%)	379 (62·7)	545 (68·2)	0·04
Comorbidity				
Hypertension	N (%)	212 (35·1)	287 (35·9)	0·79
Diabetes	N (%)	115 (19)	174 (21·8)	0·24
Laboratory Data				
PaO_2_	mmHg	58 (49–69)	53 (43–62·8)	<0·001
PaCO_2_	mmHg	35 (31–38)	33 (30–38)	0·003
PaO_2_/FiO_2_	mmHg	275 (231·2–318·5)	245 (199–289)	<0·001
pH		7·48 (7·45–7·51)	7·49 (7·46–7·51)	0·01
Lactate	mmol*L^−1^	1 (0·7–1·5)	1·1 (0·8–1·5)	0·16
Leukocytes	10^9^*L^−1^	6·8 (5·1–9·1)	7·7 (5·8–10·5)	<0·001
Lymphocytes	10^9^*L^−1^	1 (0·7–1·4)	0·9 (0·7–1·2)	0·01
Platelets	10^9^*L^−1^	171 (129–226)	190 (144–256)	<0·001
C-reactive protein	mg*L^−1^	79·7 (32·5–143·2)	119·7 (56·7–184·5)	<0·001
Lactate Dehydrogenase	U*L^−1^	425·5 (276–548·5)	479 (363–678)	0·12

Demographics, comorbidities and laboratory data of study subjects. Factor variables are expressed as count (%), continuous variables as median (1st - 3rd quartiles).

Primary and secondary outcomes in the enoxaparin cohort and in the no enoxaparin cohort are reported in Table 2. Thrombotic (pulmonary embolism, venous thromboembolism, acute myocardial infarction and cerebral infarction) events were more frequent in the enoxaparin group (*p* value < 0·001) and hemorrhagic events were not significantly different between the two cohorts, as summarized in Table 3.

**Table 2:** Outcomes in the Enoxaparin cohort and no-Enoxaparin cohort.

		No-Enoxaparin treatment	Enoxaparin	p value
In-hospital mortality	N (%)	154 (25·5)	200 (25)	0·98
ICU admission	N (%)	74 (11)	72 (10·4)	0·79
Hospital length of stay	days	5 (3–7)	9 (6–15)	<0·001

Factor variables are expressed as count (%), continuous variables as median (1st - 3rd Quartiles).

**Table 3:** Thrombotic and hemorrhagic events.

		No Enoxaparin treatment	Enoxaparin (prophylactic)	Enoxaparin (therapeutic)	p value
Patients	N (%)	604 (43)	487 (35)	312 (22)	
Thrombotic events	N (%)	13 (2·2)	12 (2·5)	51 (16)	<0·001
Pulmonary Embolism		1	3	29	
Venous thromboembolism		2	1	14	
Acute myocardial infarction		6	4	6	
Cerebral infarction		4	4	2	
Hemorrhagic events	N (%)	15 (2·5)	6 (1·2)	10 (3·2)	0·12

Recorded thrombotic and hemorrhagic events in the two cohorts. Patients in the enoxaparin cohort are divided according to dosage of received: prophylactic ≤ 40 mg a day or therapeutic > 40 mg a day. P values were computed with Fisher's exact test.

### 3.1 Primary outcome

Patient demographics, comorbidities, and laboratory parameters between patients who survived and those who did not were compared (Table 4).

**Table 4:** Patients characteristic of survivors and non-survivors at hospital discharge.

		Survivors	Non-survivors	*p* value
Patients	N (%)	1022 (74·3)	354 (25·7)	
Age	years	68 (57–76)	77 (71–83)	<0·001
BMI	kg*m^−2^	26 (24–29)	26 (24–29)	0·11
Gender				
Male	N (%)	648 (63·4)	261 (73·7)	0·001
Comorbidity				
Hypertension	N (%)	377 (36·9)	122 (34·5)	0·45
Diabetes	N (%)	204 (20)	85 (24)	0·12
Laboratory Data				
PaO_2_	mmHg	57 (49–67·2)	47 (38–56)	<0·001
PaCO_2_	mmHg	34 (31–38)	33 (29·5–38)	0·003
PaO_2_/FiO_2_	mmHg	270·5 (231–313·8)	210·5 (162–254·8)	<0·001
pH	mmol*L^−1^	7·49 (7·46–7·51)	7·48 (7·43–7·51)	<0·001
Lactate	mmol*L^−1^	1 (0·7–1·3)	1·3 (1- 2·1)	<0·001
Leukocytes	10^9^*L^−1^	7·2 (5·5–9·6)	7·6 (5·3–10·6)	0·15
Lymphocytes	10^9^*L^−1^	1 (0·7–1·4)	0·8 (0·6 to 1·1)	<0·001
Platelets	10^9^*L^−1^	185 (143–246)	164 (122- 226)	<0·001
C-reactive protein	mg*L^−1^	81·3 (34·5–153·5)	143·3 (91·3–198·1)	<0·001
Lactate Dehydrogenase	U*L^−1^	423 (321–616)	641 (472- 795)	0·02
Enoxaparin	N (%)	580 (56·8)	200 (56·5)	0·98
Outcomes				
ICU admission	N (%)	78 (7·6)	96 (27·1)	<0·001
Hospital length of stay	days	7 (5–11)	7 (3–11)	0·001

Demographics, comorbidities, laboratory data and outcomes of survivors and nonsurvivors. Factor variables are expressed as count (%), continuous variables as median (1st - 3rd quartiles).

Older age and male sex were associated with in-hospital mortality. Survivors were less hypoxic; serum lactate levels were lower in survivors. Platelet count was significantly lower in patients who died. Patients who died also had higher C-reactive protein and Lactate Dehydrogenase. Exposure to enoxaparin was not associated with mortality at the unadjusted analysis.

The results of the adjusted analysis are reported in Table 5. Enoxaparin was associated with lower in-hospital mortality (OR 0·53, 95% C.I. 0·40–0·70) compared with no enoxaparin treatment, E-value 2·08 (upper C.I. 1·66). Results of sensitivity analyses were in agreement with the results of the reported model. Four hundred and eighty seven patients in the enoxaparin cohort received a prophylactic dose of 40 mg of enoxaparin per day, 312 patients received a therapeutic dose of more than 40 mg of enoxaparin per day. For patients receiving a prophylactic dose of enoxaparin the estimated OR was 0·50 (95% C.I. 0·36–0·69) and for patients receiving a therapeutic dose OR was 0·54 (95% C.I. 0·38–0·76). One hundred ninety four patients received enoxaparin for 1–2 days (OR 1·41, 95% C.I. 0·96–2·08), 153 for 2–4 days (OR 0·52, 95% C.I. 0·35–0·79), and 452 for more than 4 days (OR 0·34, 95% C.I. 0·24–0·48).

**Table 5:** Results of adjusted analysis.

	Model	Enoxaparin vs No-Enoxaparin treatment
In-hospital mortality	Logit regression	0·53 (0·40–0·70)
Admission ICU	Logit regression	0·48 (0·32–0·69)
Hospital length of stay	Poisson regression	1·45 (1·36–1·54)

Odds Ratio (OR) and 95% confidence interval are reported for logit regression, Incidence Rate Ratios (IRR) and 95% confidence interval for Poisson regression.

### 3.2 Secondary outcomes

Treatment with enoxaparin was not associated with intensive care admission at univariate analysis. Moreover, enoxaparin treatment was associated with a reduced risk of intensive care admission at the adjusted analysis OR 0·48 (95% C.I. 0·32–0·69), E-value 2·26 (upper C.I. 1·69).

Hospital length-of-stay was longer for patients treated with enoxaparin, in the unadjusted analysis and adjusted analysis IRR 1·45 (95% C.I. 1·36–1·54).

## 4 Discussion

In our study, conducted during the recent outbreak of novel SARS-CoV-2 in Northern Italy, enoxaparin use was associated with reduced in-hospital mortality. This difference was present after adjusting for patient characteristics, severity of disease (PaO_2_/FiO_2_, C-reactive protein) and for other treatments received (azithromycin, hydroxychloroquine, corticosteroids). Propensity score was used to make outcomes comparable in the two cohorts, by adjusting for covariates that were differently distributed and associated with enoxaparin exposure in our cohort. To estimate the dependency of reported odds ratios on residual unmeasured confounders we used the E-value. The reported E-value of 2·08 (upper C.I. 1·66) is the minimum strength of association that an unmeasured confounder would need to have with both enoxaparin treatment and hospital survival, so as to explain away the association of enoxaparin treatment and reduced mortality (OR 0·53, 95% C.I. 0·40–0·70). This could also be interpreted as: an hypothetical unmeasured confounder would have to be related to the treatment and to the outcome with a relative risk of 2·08 (upper C.I. 1·66) to nullify the protective effect of enoxaparin [13,24]. In the post-hoc analyses the protective effect of enoxaparin was evident for patients that received the treatment for more than 2 days, and the association with a lower mortality was even stronger for patients receiving enoxaparin for more than 4 days. This finding supports the hypothesis that a longer treatment is more effective because it significantly reduces the probability of thrombotic complication.

Our evidence is in line with reports from other parts of the world, supporting the notion that patients admitted with COVID-19 are generally male and older than 65 years [14], [15], [16]. Differently for what has been described, arterial hypertension and diabetes mellitus were not associated with mortality [14], [15], [16].

Our data were recorded at the very beginning of the pandemic crisis of SARS-CoV-2, and back at that time, there were not clear recommendations on thromboprophylaxis in COVID-19. The evaluation, obtained with the Padua score, of the risks and benefits of thromboprophylaxis was the duty of the attending physician [17,18]. For these reasons, when evaluating the effect of enoxaparin, it was possible to have a large control group.

During the recent outbreak of novel coronavirus infection in Wuhan (China), significantly abnormal coagulation parameters were associated with worst prognosis in patients with severe pneumonia from SARS-CoV-2 [2]. Postmortem and clinical finding suggest that pulmonary embolism is common in COVID 19 patients [4,8]. The rational use of low molecular weight heparin (LMWH) in severe cases of COVID-19 is justified by the need to control thromboembolic phenomena, that may be the consequence of the hyperinflammatory status, and its complication as pulmonary embolism [4,19], which can aggravate the ventilation perfusion mismatch and can be the physiopathological explanation for the profound hypoxia in COVID-19 patients. In fact SARS-CoV-2 infection is frequently associated with the release of proinflammatory cytokines . This results in a systemic inflammatory response syndrome, which accelerates cell death, and causes multiple organ dysfunction syndrome [3]. The inflammatory reaction damages the microvascular system and generates an abnormal activation of the coagulation system, that results in a generalised small vessel vasculitis and extensive microthrombosis. This condition has been named thromboinflammation or COVID-19-associated coagulopathy, and it can also explain the increased risk of ischemic stroke reported in this patients [19,20].

In a retrospective study conducted by Tang et al. [7] at the Tongji Hospital of Wuhan, heparin treatment was found to reduce mortality in subjects affected by severe COVID-19 who experienced sepsis-induced coagulopathy. Moreover, among subjects who did not receive heparin, mortality seemed to increase along with D-dimer levels. Furthermore, the decrease in mortality was not confirmed in those patients, treated with enoxaparin, who did not experience the hypercoagulable state induced by the infection.

Ayerbe et al. [21] collected data from patients with COVID-19 admitted to seventeen Spanish hospitals. They confirmed that heparin treatment was associated with lower mortality. nonetheless, since from their data it was possible to adjust the analysis for only two variables, the effect of unmeasured confounder could be substantial.

A different study was conducted among patients with laboratory-confirmed COVID-19 hospitalized within the Mount Sinai Health System in New York City [22], who received systemic anticoagulation (including oral, subcutaneous, or intravenous forms) during their hospital course. In these patients, administration and duration of treatment with anticoagulants were associated with reduced mortality.

In the present study we confirmed these findings, since treatment with enoxaparin is associated with a better prognosis in patients admitted to our hospital with diagnosis of COVID-19. Furthermore, to our knowledge this is the first study in which enoxaparin is associated with reduced risk of intensive care admission. In the pre-proof report by Paranjpe et al., anticoagulation is associated with an increased risk of mechanical ventilation, however it is not clear how exposure to enoxaparin was recorded when evaluating the risk of admission to intensive care [22]. In fact, due to the gravity of their disease, it is very common for all patients in intensive care to receive thrombotic prophylaxis [23]. This could generate a bias, if hospital exposure to enoxaparin is used, leading to the conclusion that enoxaparin increases the risk of mechanical ventilation. Instead, we were able to record exposure to enoxaparin in the hospital ward to estimate the risk of intensive care admission.

As can be seen in Table 3, there was a higher frequency of patients with thrombotic events in the enoxaparin group. This is reasonably due to the fact that patients with thrombotic events were treated with enoxaparin. Nonetheless, enoxaparin use was associated with a lower mortality rate despite the fact that thrombotic events were distributed unevenly.

The main limitation of our study is related to its retrospective observational nature: the absence of randomization makes it impossible to exclude the presence of bias or unmeasured confounders. Nonetheless, even if randomized clinical trials (RCTs) are the gold standard in assessing a treatment's efficacy and safety, observational studies, which usually have a greater external validity than RCTs, can add information on the use of certain drugs in the clinical field and should be integrated with RCTs. In conclusion, this retrospective observational study shows that treatment with enoxaparin during hospital stay was associated with a lower death rate, therefore it supports the use of enoxaparin in all patients admitted to hospital for COVID-19. Moreover, when enoxaparin is used on the wards, it reduces the risk of ICU admission. Generalization of this finding to other low molecular weight heparins will likely be possible as soon as more evidence from observational studies or randomized clinical trials is available, since different low molecular weight heparins have been shown to share thrombo-prophylactic efficacy and bleeding risk.

Since the usefulness of enoxaparin in improving the clinical outcome in COVID-19 patients seems consistent, and its use is already routine in many hospital wards and intensive care units, there is urgent need for randomized trials to evaluate their clinical efficacy and safety in patients with infection from SARS-CoV-2.

## Declaration of Competing Interest

The authors declare that they have no known competing financial interests or personal relationships that could have appeared to influence the work reported in this paper.
